# Determination of Standardized Ileal Amino Acid Digestibilities in Different Soybean Meals for Yellow-Feathered Chickens and Development of Prediction Models

**DOI:** 10.3390/ani16010089

**Published:** 2025-12-28

**Authors:** Qianwen Yuan, Wenpeng Chen, Jiali Long, Liyang Zhang, Shengchen Wang, Tingting Li, Yun Hu, Xiaoyan Cui, Xugang Luo

**Affiliations:** 1 Poultry Mineral Nutrition Laboratory, College of Animal Science and Technology, Yangzhou University, Yangzhou 225000, China; 18452563770@163.com (Q.Y.); mz120221519@stu.yzu.edu.cn (W.C.); mx120240937@stu.yzu.edu.cn (J.L.); shengchenwang@yzu.edu.cn (S.W.); 007174@yzu.edu.cn (T.L.); huyun@yzu.edu.cn (Y.H.); 2 Mineral Nutrition Research Division, State Key Laboratory of Animal Nutrition and Feeding, Institute of Animal Science, Chinese Academy of Agricultural Sciences, Beijing 100193, China; zhangliyang@caas.cn

**Keywords:** soybean meal, medium-growing yellow-feathered chicken, standardized ileal amino acid digestibility, prediction model

## Abstract

Currently, there is no report on prediction models of standardized ileal amino acid digestibilities (SIAADs) in soybean meals (SBMs) for medium-growing yellow-feathered chickens. This study firstly analyzed the chemical compositions of 10 SBMs, then determined their SIAADs in chickens, and finally established and verified prediction models for SBM SIAADs based on their chemical compositions and amino acid (AA) profiles. A total of 276 55 d-old Tianluma roosters were selected and randomly divided by body weight into 11 treatment groups. On d 63, chickens were fed either a nitrogen-free diet (NFD) or one of 10 SBM diets for 5 d. On d 67, ileal chyme samples were collected to determine SIAADs. Data from nine SBM samples and stepwise regressions were employed to build prediction models, while one SBM sample was randomly selected to validate model accuracy. Different SBM sources affected (*p* ≤ 0.007) SIAADs in medium-growing yellow-feathered chickens. The standardized ileal digestibility (SID) of glutamic acid (Glu) was the highest (93.9%), whereas that of cysteine (Cys) was the lowest (81.7%). Fifteen prediction models (*R*^2^ = 0.567–0.993, *p* < 0.03) for the SIDs of methionine (Met), isoleucine (Ile), leucine (Leu), phenylalanine (Phe), lysine (Lys), histidine (His), arginine (Arg), aspartic acid (Asp), serine (Ser), Glu, glycine (Gly), alanine (Ala), Cys, tyrosine (Tyr), and proline (Pro) in SBMs for medium-growing yellow-feathered chickens were effectively established based on chemical compositions and AA profiles. Among them, the prediction model for the SID of Cys showed the best fit (*R*^2^ = 0.993, *p* = 0.002), while the model for the SID of Ala had the lowest fit (*R*^2^ = 0.567, *p* = 0.019). Except for His and Pro, which exhibited poor predictive accuracy, all other models showed good accuracy. These prediction models thus provide a valuable reference for rapidly estimating the SIDs of key AAs in SBMs for medium-growing yellow-feathered chickens.

## Simple Summary

In view of our country’s high dependence on soybean meal (SBM) imports, the fact that feed costs account for 60–70% of the total costs of broiler production, and the current situation in which most standardized ileal amino acid digestibility (SIAAD) data were derived from white-feathered broilers with a lack of specific data for medium-growing yellow-feathered chickens, the present study systematically determined the SIAAD values of SBMs from different sources for this chicken variety, and established rapid prediction models for SIAADs in SBM. The results provide a scientific basis for the precise feeding of medium-growing yellow-feathered chickens, significantly improving feed utilization efficiency, reducing breeding costs, and contributing to the sustainable development of animal husbandry.

## 1. Introduction

Soybean meal (SBM), a by-product of soybean oil extraction, is the most commonly used protein feed ingredient and is characterized by high protein content, high nutritional value, good palatability, and high amino acid digestibility in broilers. With the rapid development of intensive broiler production, the demand for SBM has been increasing rapidly. The rising price of SBM has markedly increased the cost of broiler production. The nutritional value of SBM mainly derives from its crude protein (CP) and indispensable amino acid (AA) contents. These differences are largely driven by soybean cultivar/genetic makeup and environmental conditions in the production areas, such as temperature, precipitation, and soil properties, which directly affect the nutritional value of soybeans [1,2,3,4]. In addition, processing technology is another key factor. Among the processing methods, heat treatment is most widely applied, including extrusion and steam treatment. Romarheim et al. [5] demonstrated that extrusion can effectively inactivate trypsin inhibitor (TI) in SBM, thereby significantly improving protein digestibility. Moreover, both the temperature and duration of steam treatment are important parameters that determine SBM quality, as they not only affect protein quality but also directly determine the residual level of TI, the main anti-nutritional factor [6]. Espinosa et al. [7] reported that autoclaving SBM for 3 to 25 min improved broiler growth performance by effectively degrading TIs. Therefore, to accurately evaluate the nutritional utilization of different SBMs in broilers, it is essential to determine their nutritional values and establish reliable prediction models.

The AAs are essential nutrients for broilers, playing vital roles in regulating diverse physiological functions, including growth, feed conversion efficiency, and health [8,9,10]. However, the intensive development of animal husbandry has contributed to increased nitrogen emissions and environmental pollution. Kriseldi et al. [11] showed that reducing dietary CP levels through AA supplementation significantly reduced nitrogen emissions. Other studies have shown that insufficient or excessive dietary CP and AA levels can reduce production performance and further increase nitrogen emissions [12,13]. Therefore, accurate evaluation of AA availabilities in feed ingredients is critical for precise AA supplementation. Apparent ileal amino acid digestibility (AIAAD) is widely used to determine the concentration of digestible AAs in poultry diets [14], but it does not account for basal endogenous losses (BELs) of AAs from the gastrointestinal tract [15]. In contrast, standardized ileal amino acid digestibility (SIAAD) accounts for these inevitable losses. Studies have shown that SIAAD values are more additive than AIAAD values, and thus the use of SIAAD values in feed formulation can greatly improve formulation accuracy and practicality [16,17,18]. Consequently, SIAAD is considered more suitable for assessing AA utilization in poultry [19]. Recently, Sheikhhasan et al. [20] evaluated SIAADs in nine different SBMs for fast-growing white-feathered broilers and successfully established prediction models based on chemical composition. However, these models cannot be directly applied to medium-growing yellow-feathered chickens majorly due to significant differences in SIAAD values of SBMs between broiler breeds [20,21]. The SIAAD values in SBMs were reported to be consistently and significantly higher for fast-growing white-feathered broilers [20] than for yellow-feathered chickens [21] [90.3 vs. 79.9% for valine (Val), 90.2 vs. 83.3% for methionine (Met), 91.7 vs. 79.7% for isoleucine (Ile), 91.7 vs. 80.2% for leucine (Leu), 90.2 vs. 78.6% for threonine (Thr), 92.2 vs. 80.3% for phenylalanine (Phe), 91.8 vs. 76.0% for lysine (Lys), 91.8 vs. 82.6% for histidine (His), 94.1 vs. 84.9% for arginine (Arg), 89.2 vs. 77.6% for aspartic acid (Asp), 90.0 vs. 80.4% for serine (Ser), 93.2 vs. 80.8% for glutamic acid (Glu), 86.5 vs. 77.3% for glycine (Gly), 90.2 vs. 80.7% for alanine (Ala), 80.7 vs. 76.6% for cysteine (Cys), and 89.5 vs. 78.1% for proline (Pro)]. This means that using the SIAADs-predicting models of SBMs for fast-growing white-feathered broilers would over-predict the SIAAD values of SBMs for yellow-feathered broilers. Therefore, it is necessary to establish SIAAD prediction models of SBMs for yellow-feathered chickens. However, to date, no study on this aspect has been reported in yellow-feathered chickens.

Based on this gap, we hypothesized that prediction models for SIAADs in SBMs for medium-growing yellow-feathered chickens could be established using their chemical compositions and AA profiles. To test this hypothesis, we firstly determined the contents of conventional nutrients and anti-nutritional factors in 10 different SBMs, then evaluated their SIAADs in medium-growing yellow-feathered chickens, established prediction models based on their chemical compositions and AA profiles, and finally verified the accuracy of these models.

## 2. Materials and Methods

### 2.1. Animal Ethics

The care and use of animals complied with the guidelines of the Animal Use Committee of the Chinese Ministry of Agriculture (Beijing, China). The ethics application was approved by the Ethics Committee of the Department of Animal Science and Technology at Yangzhou University (permit number: SYXK (Su) 2021-0027).

### 2.2. Experimental Design and Treatments

In the present study, a completely randomized design was adopted. A total of 11 dietary treatments were designed, including 1 nitrogen-free diet (NFD) group and 10 SBM diet groups.

### 2.3. SBMs, Animals and Diets

Ten tested SBM samples (numbered A1–A10) were obtained from suppliers in different places. The original information for the 10 SBM samples was shown in Table 1.

A total of 300 55 d-old Tianluma roosters (Huai’an Wen’s Livestock Co., Ltd., Huai’an, China) were housed in stainless steel cages (90 × 70 × 45 cm, providing a stocking density of 10 birds/m^2^ for the NFD group and 6 birds/m^2^ for the SBM groups) equipped with feeders and drinkers, with the environment maintained at 21–25 °C and 50–55% relative humidity. On d 60, chickens were weighed after fasting to ensure body weights matched the reference standard for yellow-feathered chickens of this age. Subsequently, 276 chickens with similar weights (averaging 1.36 kg/bird) were randomly assigned to 11 treatment groups. Each group was allocated to six replicate cages, with four birds per replicate for the SBM diets and six for the NFD group (to compensate for its very low feed intake and ensure sufficient terminal ileal digesta collection for analyses). From 55 to 62 d of age, all chickens were fed ad libitum the same commercial complete pelleted diet and tap water (Huai’an Wen’s Livestock Co., Ltd., Huai’an, China). From 63 to 67 d of age, birds were fed ad libitum the NFD and SBM diets in pellet form and tap water. Tianluma roosters were selected as a representative model for medium-growing yellow-feathered chickens for the following reasons: firstly, the Tianluma breed is a variety from Wen’s Group, a leading livestock company in China, which has considerable influence in the industry. Secondly, the Tianluma breed is commercially dominant in China, holding a significant market share, which ensures the practical relevance and application of our findings. Thirdly, Tianluma roosters possess a series of superior traits that make them an ideal subject for research. They exhibit robust growth potential, excellent feed conversion ratio, and uniform body size, which guarantees the reliability and repeatability of experimental data. And fourthly, this breed features strong disease resistance and high survival rates, reflecting its sound genetic foundation and robust constitution.

The NFD was formulated using corn starch, glucose, cellulose, and a micronutrient premix. The 10 SBM diets were prepared using SBM, corn starch, glucose, and a micronutrient premix, with SBM as the sole source of CP and AAs. Titanium dioxide (TiO_2_) was added to both the SBM diets and the NFD at 0.4% as an indigestible marker. All diets were formulated to meet the nutrient requirements except for AAs for yellow-feathered broilers at the corresponding stages (NY/T 3645-2020) [22]. The analyzed chemical compositions of the 10 SBMs are shown in Table 2, and the formulations and nutrient levels of 11 diets are presented in Table 3.

### 2.4. Sample Collections and Preparations

SBM samples were collected for analyses of conventional nutritional components, anti-nutritional factors, and AA contents. Diets were collected for assays of CP and AA contents. SBMs and diets were ground through a 0.45 mm sieve by using a stainless-steel grinding mill (Yanshanzhengde Inc., Beijing, China) to facilitate analyses.

On d 67, all chickens were euthanized by carbon dioxide asphyxiation in a fed state. Chyme samples were immediately collected from the posterior 1/2 of the ileum in a folded fashion (with the final 2 cm discarded) and stored at −20 °C. The frozen chyme was then freeze-dried and rehydrated at room temperature for 24 h. The lyophilized chyme from all chickens in each replicate cage was pooled and ground to obtain homogeneous samples for subsequent analyses.

### 2.5. Measurement Indicators and Methods

Moisture, CP, gross energy (GE), ether extract (EE), crude fiber (CF), and crude ash (CA) contents in SBMs and CP in diets were determined according to the official methods of AOAC [23]. The neutral detergent fiber (NDF) and acid detergent fiber (ADF) in SBMs were measured according to the methods described by Yun et al. [24]. TI in SBMs was measured according to the method of Hamerstrand et al. [25]. Phytic acid (PA) content in SBMs was determined using a commercial kit (catalog number SAP-2-Y; Jiangsu Addison Biotechnology Co., Ltd., Suzhou, China). The contents of AAs in SBMs, diets, and chymes were determined according to the method of AOAC [23]. TiO_2_ contents in diets and chymes were analyzed according to the method of Titgemeyer et al. [26].

### 2.6. Calculations

The BEL, AIAAD, and SIAAD were calculated according to the following formulas:

BEL (mg/kg) = (W_I_ × W_II_)/W_III_

AIAAD (%) = 100 − (D_I_ × D_II_)/(D_III_ × D_IV_) × 100

SIAAD (%) = AIAAD + BEL/D_III_ × 100

where all data were expressed on a DM basis. W_I_ = AA concentration (mg/kg) in the chyme of broilers fed the NFD, W_II_ = TiO_2_ concentration (mg/kg) in the NFD, W_III_ = TiO_2_ concentration (mg/kg) in the chyme of broilers fed the NFD, D_I_ = AA concentration (mg/kg) in the chyme of broilers fed SBM diets, D_II_ = TiO_2_ concentration (mg/kg) in SBM diets, D_III_ = AA concentration (mg/kg) in SBM diets, D_IV_ = TiO_2_ concentration (mg/kg) in the chyme of broilers fed SBM diets.

### 2.7. Statistical Analyses

A one-way ANOVA was performed on AIAAD and SIAAD data using the GLM procedure in SAS (2013, version 9.4; SAS Institute Inc., Cary, NC, USA). Significant differences among treatments were evaluated using the LSD method [27]. Pearson correlation analysis was used to examine the correlation relationships between chemical compositions, AA profiles, and SIAADs of SBMs. Data from nine SBM samples (A1–A3, A5–A10) were used in stepwise regression to develop SIAAD prediction models. The RANDGEN function in SAS was applied to randomly select one SBM sample (A4) for model validation. Each replicate cage served as the experimental unit, and significance was set at *p* < 0.05.

## 3. Results

### 3.1. Chemical Compositions of 10 Tested SBM Samples

The chemical compositions, as detailed in Table 2 (air-dry basis), showed values of 17.13 to 17.50 MJ/kg for GE, 8.94 to 12.99% for moisture, 43.15 to 47.10% for CP, 0.79 to 1.61% for EE, 4.02 to 10.91% for CF, 9.78 to 21.73% for NDF, 5.24 to 12.01% for ADF, with the contents of CA, nitrogen-free extract (NFE), PA, and TI falling between 6.05–7.60%, 24.45–30.12%, 8.01–13.80 g/kg, and 8.38–17.13 g/kg, respectively. Among them, the coefficient of variation (CV) of GE was the lowest (0.75%), whereas that of CF was the highest (32.32%). The contents of essential amino acids (EAAs) Val, Met, Ile, Leu, Thr, Phe, Lys, His, Arg, and tryptophan (Trp) ranged from 2.23 to 2.45%, 0.52 to 0.59%, 2.05 to 2.27%, 3.59 to 3.96%, 1.89 to 2.05%, 2.43 to 2.72%, 2.94 to 3.19%, 1.22 to 1.33%, 3.40 to 3.69%, and 0.52 to 0.61%, respectively. The contents of non-essential amino acids (NEAAs) Asp, Ser, Glu, Gly, Ala, Cys, tyrosine (Tyr), and Pro ranged from 5.29 to 5.82%, 2.46 to 2.67%, 8.44 to 9.31%, 2.01 to 2.15%, 2.02 to 2.19%, 0.40 to 0.47%, 1.50 to 1.74%, and 2.48 to 2.69%, respectively. The CVs of EAAs ranged from 2.89 to 4.76%, with Trp showing the greatest variability and Thr the least. For NEAAs, CVs ranged from 2.23 to 6.27%, with Cys showing the highest variability and Gly the lowest.

### 3.2. AIAADs of 10 Different Sources of SBMs for Medium-Growing Yellow-Feathered Chickens

As shown in Table 4, different sources of SBMs affected (*p* ≤ 0.003) the apparent ileal digestibilities (AIDs) of 15 AAs except for Ala, Cys, and Tyr in medium-growing yellow-feathered chickens. For EAAs, the AID values of Val, Met, Ile, Leu, Thr, Phe, Lys, His, Arg, and Trp were 80.0–86.6%, 82.9–89.3%, 84.3–87.7%, 87.0–90.4%, 74.5–80.6%, 83.4–89.7%, 82.9–87.4%, 80.1–87.1%, 88.7–91.4%, and 76.3–85.4%, respectively. Among them, Arg showed the highest AID (90.0%), whereas that of Thr was the lowest (77.0%). For NEAAs, the AID values of Asp, Ser, Glu, Gly, Ala, Cys, Tyr, and Pro were 80.0–88.2%, 81.9–86.9%, 87.9–92.7%, 75.7–83.0%, 78.4–82.7%, 68.9–74.0%, 81.6–85.7%, and 71.1–79.7%, respectively. Among them, Glu had the highest AID (90.6%), while that of Cys was the lowest (72.1%). The CVs of EAAs were 2.43% for Val, 1.94% for Met, 1.20% for Ile, 1.31% for Leu, 2.78% for Thr, 2.44% for Phe, 1.40% for Lys, 2.54% for His, 1.05% for Arg, and 3.27% for Trp. Among them, Arg showed the most stable AID, while that of Trp was somewhat less stable. The CVs of NEAAs were 3.13% for Asp, 2.08% for Ser, 1.50% for Glu, 2.46% for Gly, 1.89% for Ala, 1.83% for Cys, 1.37% for Tyr, and 3.56% for Pro. Among them, Tyr exhibited the lowest variability, while that of Pro was the highest.

### 3.3. SIAADs of 10 Different Sources of SBMs for Medium-Growing Yellow-Feathered Chickens

As shown in Table 5, different sources of SBMs affected (*p* ≤ 0.007) the standardized ileal digestibilities (SIDs) of 15 AAs except for Ala, Cys, and Tyr in medium-growing yellow-feathered chickens. For EAAs, the SID values of Val, Met, Ile, Leu, Thr, Phe, Lys, His, Arg, and Trp were 86.8–92.3%, 86.2–92.9%, 88.9–91.8%, 91.0–94.8%, 82.8–88.8%, 88.7–94.6%, 86.3–90.4%, 83.2–89.5%, 92.3–94.8%, and 81.8–89.8%, respectively. Among them, Arg showed the highest SID (93.8%), whereas that of Trp was the lowest (84.5%). For NEAAs, the SID values of Asp, Ser, Glu, Gly, Ala, Cys, Tyr, and Pro were 84.3–92.3%, 87.8–92.3%, 91.2–96.0%, 81.3–87.2%, 83.9–87.3%, 80.2–82.7%, 84.6–88.4%, and 81.1–88.8%, respectively. Among them, the SID of Glu was the highest (93.9%) and that of Cys the lowest (81.7%). The CVs of EAAs were 1.98% for Val, 1.89% for Met, 1.06% for Ile, 1.26% for Leu, 2.19% for Thr, 2.14% for Phe, 1.22% for Lys, 2.32% for His, 0.75% for Arg, and 2.67% for Trp. Among them, Arg showed the most stable SID, while that of Trp was somewhat less stable. The CVs of NEAAs were 2.91% for Asp, 1.72% for Ser, 1.56% for Glu, 1.92% for Gly, 1.47% for Ala, 0.94% for Cys, 1.27% for Tyr, and 2.87% for Pro. Among them, Cys exhibited the lowest variability, while that of Asp was the highest.

### 3.4. Correlations Between Chemical Compositions and SIAADs of SBMs for Medium-Growing Yellow-Feathered Chickens

As shown in Table 6, the SIDs of Arg and Gly were positively correlated (*p* < 0.05) with GE content. The SID of Ile was positively correlated (*p* < 0.05) with CP content. In contrast, the SIDs of Phe, Asp, and Pro were negatively correlated (*p* < 0.05) with EE content. A negative correlation (*p* < 0.05) was observed between the SIDs of Ile and CF and ADF content. The SIDs of Phe, Lys, His, and Cys were negatively correlated (*p* < 0.05) with NDF content. The SIDs of Leu, Phe, and Asp were positively correlated (*p* < 0.05) with CA content. Additionally, the SIDs of Leu, Glu, and Ala were negatively correlated (*p* < 0.05) with PA content, and the SID of Glu was also negatively correlated (*p* < 0.05) with NFE content. All other positive or negative correlations were not significant.

### 3.5. Correlations Between AA Profiles and SIAADs of SBMs for Medium-Growing Yellow-Feathered Chickens

As shown in Table 7, the SID of Ile was positively correlated (*p* < 0.05) with the contents of several EAAs, including Val, Ile, Leu, Thr, Lys, His, and Arg. The SIDs of both Leu and Ser were positively correlated (*p* < 0.05) with Met content. As shown in Table 8, the SID of Ile was also positively correlated (*p* < 0.05) with the contents of multiple NEAAs, including Asp, Ser, Glu, Gly, Ala, and Pro. All other positive or negative correlations were not significant.

### 3.6. Prediction Models for SIAADs of SBMs in Medium-Growing Yellow-Feathered Chickens Based on Their Chemical Compositions and AA Profiles

The established prediction models for SBM SIAADs in medium-growing yellow-feathered chickens are shown in Table 9. In total, 15 predicting models were effectively developed (*R*^2^ = 0.567–0.993, *p* < 0.03) based on their chemical compositions and AA profiles. The *R*^2^ values of the regression models for the SIDs of Met, Ile, Leu, Phe, His, Asp, Ser, and Cys were all more than 0.90. The model for the SID of Cys had the best fit (*R*^2^ = 0.993, *p* = 0.002), whereas the model for the SID of Ala had the poorest fit (*R*^2^ = 0.567, *p* = 0.019).

### 3.7. Verification of the Accuracy of SIAADs Prediction Models for SBMs

To validate prediction accuracy, the chemical composition of one SBM sample (A4) was substituted into the models. The predicted SID values for Met, Ile, Leu, Phe, Lys, His, Arg, Asp, Ser, Glu, Gly, Ala, Cys, Tyr, and Pro in SBM A4 were obtained as shown in Table 10. Differences between measured and predicted values of the above AAs in SBM A4 were 1.7%, 0.1%, 0.6%, 1.2%, 1.1%, 4.5%, 0.8%, 1.7%, 1.5%, 0.7%, 1.1%, 1.3%, 2.2%, 0.2%, and 5.7%, respectively. For all other AAs except His and Pro, predicted values fell within the ranges of measured values ± standard deviations (SDs), indicating good predictive accuracies.

## 4. Discussion

In the present study, the chemical compositions and the SIDs of Val, Met, Ile, Leu, Thr, Phe, Lys, His, Arg, Trp, Asp, Ser, Glu, Gly, Ala, Cys, Tyr, and Pro were determined in 10 SBM samples for medium-growing yellow-feathered chickens. Corresponding SIAAD prediction models were then established and validated. The results demonstrate that chemical compositions (CP, EE, CA, ADF, NFE, NDF, PA, GE, and TI) and AA profiles (Cys, Ala, Ser, Trp, Arg, Met, Phe, and Lys) in SBMs could be effectively used to develop prediction models for most AAs in medium-growing yellow-feathered chickens except for His and Pro, because the predicted SID values for His and Pro fell outside the determined values ± SDs, and the predicting models for the SIDs of Val, Thr, and Trp could not be effectively established. To date, although the construction of SIAAD prediction models of SBMs based on their chemical compositions has been reported in fast-growing white-feathered broilers and pullets [20,28], no study on this aspect is available in yellow-feathered chickens. Therefore, the present study filled this critical gap. These findings support our initial hypothesis and provide a scientific basis for rapidly predicting SIAADs in SBMs and for formulating precise AA supplementation strategies in medium-growing yellow-feathered chickens.

The chemical compositions of SBMs vary due to factors such as soybean cultivar, growing environment, and processing conditions [29,30,31]. Yang et al. [32] analyzed 10 SBM samples from nine provinces in China and reported slightly higher GE contents than those observed in our study, possibly due to different sources of SBMs. Lagos and Stein [33] analyzed 24 SBM samples from China, Argentina, Brazil, USA, and India. Their reported CP, ash, EE, ADF, Val, Ile, Leu, Thr, Phe, Lys, His, Arg, Asp, Ser, Glu, Gly, Tyr, Ala, and Pro contents were generally similar to our results, although their PA, Met, Trp, and Cys levels were somewhat higher and their NDF levels slightly lower. The reason for the above discrepancies may be that the SBM sample size in the current study was relatively small. The present study revealed lower variations (CVs < 10%) in the GE, CP, CA, NFE, Val, Met, Ile, Leu, Thr, Phe, Lys, His, Arg, Trp, Asp, Ser, Glu, Gly, Ala, Cys, Tyr, and Pro contents of the 10 SBM samples. In contrast, elevated levels of variability (CVs > 10%) were exhibited by moisture (13.03%), EE (20.89%), CF (32.32%), NDF (25.70%), ADF (30.46%), PA (20.05%), and TI (17.90%). A comparison with existing research data on the variations in SBM compositions across different regions and varieties showed that the CVs for most chemical compositions in the 10 SBMs analyzed in the current study were similar to those reported in previous studies [19,28,34]. The significant variations in chemical composition across regions and varieties confirmed their diversity, making the data suitable for building precise predictive models.

The AIAAD values do not account for BELs and then lead to inaccurate estimates [35,36]. The present study revealed a relatively wide variation in the AIAADs of SBMs, with ranges of 80.0–86.6% for the AID of Val, 74.5–80.6% for the AID of Thr, 76.3–85.4% for the AID of Trp, 80.0–88.2% for the AID of Asp, and so on. The above observed variations can be attributed to the fact that AID values are susceptible to the influence of BELs. In contrast, SIAAD values provide a more accurate estimation of the true availabilities of AAs in feedstuffs, as the SIAADs eliminate the interferences from BELs. De Coca-Sinova et al. [37] reported that AIAAD values for SBMs were similar to our results for Val and Ala but lower for Arg, His, Ile, Leu, Lys, Met, Thr, Asp, Cys, Glu, Gly, and Ser, while slightly higher for Phe and Ala. These differences may reflect variations in chicken breeds, rearing conditions, and feed batches. Because SIAADs correct for BELs, they more accurately reflect AA utilization and are thus critical for precise dietary AA formulation to minimize feed costs and nitrogen emissions [38]. Our present results show that different sources of SBMs significantly affected the SID values of Val, Met, Ile, Leu Thr, Phe, Lys, His, Arg, Trp, Asp, Ser, Glu, Gly, Ala, Cys, Tyr, and Pro for medium-growing yellow-feathered chickens. Liu et al. [21] evaluated SIAADs in one SBM for 105 d-old yellow-feathered chickens, reporting lower SID values for Val, Met, Ile, Leu, Thr, Phe, Lys, His, Arg, Asp, Ser, Glu, Gly, Cys, Ala, and Pro compared with our results. Trp and Tyr were not analyzed by Liu et al. [21], preventing direct comparison. Sheikhhasan et al. [20] also evaluated SIAADs in nine SBMs for white-feathered broilers, reporting higher SID values for Thr, Lys, His, Gly, Ala, Tyr, and Pro compared with our results. The SID values for Val, Met, Ile, Leu, Phe, Arg, Asp, Ser, Glu, and Cys were similar between the two studies. Trp was not analyzed by Sheikhhasan et al. [20], preventing direct comparison. These discrepancies likely resulted from differences in chicken breeds and age, SBM origins and sample number, and physiological status of broilers during different trials.

Establishing SIAAD prediction models for medium-growing yellow-feathered chickens represents an important step toward rapid assessment of SBM quality and precision AA supplementation. In our study, SIAADs were significantly correlated with the chemical compositions and AA profiles of SBMs. For instance, the SIDs of Arg and Gly were positively correlated with GE, suggesting that higher energy availability may promote AA utilization. The SID of Ile was positively correlated with CP but negatively correlated with CF and ADF, while the SIDs of Phe, Lys, His, and Cys were negatively correlated with NDF. These findings were consistent with the evidence that dietary fiber stimulated mucus secretion and increased endogenous AA losses [39,40,41]. Similarly, PA negatively correlated with the SIDs of Leu, Glu, and Ala, in line with previous findings that PA irritated the gastrointestinal mucosa and increased mucin secretion, thereby exacerbating endogenous AA losses [42,43]. The SID of Glu was negatively correlated with both NFE and PA, further indicating the inhibitory effects of antinutritional factors on AA digestibility. It is noteworthy that the relationship between chemical composition and digestibility may be influenced by some other factors, such as the different origins and processing conditions of SBM samples. Oviedo-Rondón et al. [44] demonstrated that SBM nutrient composition, AA digestibility, and energy content were influenced by the country of origin and year of harvest. It is reported that thermal processing conditions affected AA digestibility in SBMs [45,46], likely because of formation of indigestible complexes of AA with fiber [47]. Our SBM samples originated from different countries of origin and were processed under different conditions, leading to greater variations in their compositions and SIAADs. Additionally, the lack of effects of SBM source on SIDs of couple AAs (Ala, Cys and Tyr) was also observed in the current study, which is similar to the report of Sheikhhasan et al. [20] in fast-growing white-feathered broilers. However, specific reasons for the above lack of effects are unclear, and need a further study.

Furthermore, in the present study, 15 prediction models were established based on their chemical compositions (CP, EE, CA, ADF, NFE, NDF, PA, GE, and TI) and AA profiles (Cys, Ala, Ser, Trp, Arg, Met, Phe, and Lys), with *R*^2^ values ranging from 0.567 to 0.993. Models for the SIDs of Met, Ile, Leu, Phe, His, Asp, Ser, and Cys all had *R*^2^ > 0.90, indicating high predictive reliability. Previous research on prediction models has focused mainly on pigs [48] and fast-growing white-feathered broilers [20]. Recently, Cao et al. [28] also developed prediction models for hens based on SBM compositions. Their identification of GE as a positive predictor, CF and NDF as negative predictors corroborates our findings. Taken together, these results highlight that while antinutritional factors such as PA and dietary fiber inhibit AA utilization, higher GE and CP contents enhance AA digestibility.

To assess the accuracy of the developed SIAAD prediction models, one SBM sample (A4) was randomly selected for validation. With the exceptions of the SIDs of His and Pro, the predicted values for the SIDs of Met, Ile, Leu, Phe, Lys, Arg, Asp, Ser, Glu, Gly, Ala, Cys, and Tyr all closely matched the measured values. These agreements confirm the robustness of most models. To facilitate the application of these prediction models in medium-growing yellow-feathered chicken production, future studies should incorporate a broader range of SBM samples for validation, as the single sample used here is insufficient.

## 5. Conclusions

The prediction models for the SIDs of Met, Ile, Leu, Phe, Lys, Arg, Asp, Ser, Glu, Gly, Ala, Cys, and Tyr in SBMs for medium-growing yellow-feathered chickens were successfully established in the present study. Except for the models for the SIDs of His and Pro, which showed relatively poor predictive performances, all other models demonstrated high accuracies. Therefore, these accurate models provide a valuable reference for quickly predicting the SIAADs in SBMs for medium-growing yellow-feathered chickens based on their chemical compositions (CP, EE, CA, ADF, NFE, NDF, PA, GE, and TI) and AA profiles (Cys, Ala, Ser, Trp, Arg, Met, Phe, and Lys). Notably, the prediction models for the SIDs of Met, Ile, Leu, Phe, His, Asp, Ser, and Cys achieved R^2^ values above 0.90, indicating strong predictive performances.

## Figures and Tables

**Table 1:** Sources of the 10 tested soybean meals.

Number	Sources	Number	Sources
A1	America	A6	China
A2	Brazil	A7	America
A3	Brazil	A8	America
A4	Brazil	A9	America
A5	Brazil	A10	America

**Table 2:** Analyzed chemical compositions of the 10 tested SBM samples (air-dry basis) ^1^.

Item (%)	SBM Sources										Mean	SD	CV (%)
	A1	A2	A3	A4	A5	A6	A7	A8	A9	A10			
GE (MJ/kg)	17.32	17.13	17.20	17.31	17.13	17.33	17.19	17.40	17.43	17.50	17.29	0.13	0.75
Moisture	11.26	12.99	12.64	12.21	12.80	12.08	12.86	8.94	10.07	9.55	11.54	1.50	13.03
CP	44.63	43.15	46.81	46.54	45.28	46.79	43.47	46.27	47.10	43.32	45.34	1.58	3.49
EE	1.61	1.36	1.36	1.37	1.52	1.22	1.20	0.92	0.79	1.05	1.24	0.26	20.89
CF	6.41	9.63	4.44	4.02	7.11	7.10	10.91	6.18	5.50	9.50	7.08	2.29	32.32
NDF	13.13	21.73	15.77	12.44	17.68	11.27	14.31	9.78	10.76	17.03	14.39	3.70	25.70
ADF	6.56	12.01	6.15	5.73	8.30	6.16	9.83	5.74	5.24	9.83	7.56	2.30	30.46
CA	6.08	6.05	7.03	7.37	7.60	7.33	7.11	7.57	7.35	7.20	7.07	0.56	7.90
NFE	30.01	26.82	27.72	28.49	25.69	25.48	24.45	30.12	29.19	29.38	27.74	2.03	7.33
PA (g/kg)	13.44	13.30	8.01	9.73	9.98	9.07	8.67	11.41	13.80	9.48	10.69	2.14	20.05
TI (g/kg)	17.13	12.38	14.96	14.75	15.45	13.36	8.38	16.02	12.66	12.81	13.79	2.47	17.90
EAAs													
Val	2.32	2.27	2.45	2.39	2.30	2.38	2.23	2.31	2.41	2.27	2.33	0.07	3.02
Met	0.55	0.52	0.58	0.59	0.57	0.58	0.53	0.54	0.56	0.56	0.56	0.02	4.09
Ile	2.11	2.11	2.27	2.19	2.13	2.21	2.05	2.13	2.22	2.10	2.15	0.07	3.09
Leu	3.66	3.67	3.96	3.82	3.71	3.83	3.59	3.73	3.88	3.67	3.75	0.12	3.08
Thr	1.94	1.89	2.05	2.01	1.95	2.01	1.89	1.95	2.03	1.93	1.96	0.06	2.89
Phe	2.47	2.62	2.72	2.60	2.51	2.61	2.43	2.51	2.63	2.48	2.56	0.09	3.53
Lys	3.17	2.94	3.19	3.19	2.99	3.11	2.96	3.01	3.16	3.00	3.07	0.10	3.36
His	1.28	1.22	1.33	1.30	1.26	1.30	1.23	1.27	1.32	1.24	1.28	0.04	3.00
Arg	3.41	3.40	3.69	3.60	3.50	3.59	3.43	3.49	3.69	3.48	3.53	0.11	3.08
Trp	0.55	0.52	0.55	0.57	0.55	0.61	0.52	0.57	0.56	0.54	0.56	0.03	4.76
NEAAs													
Asp	5.44	5.36	5.82	5.62	5.44	5.63	5.29	5.48	5.70	5.40	5.52	0.17	3.01
Ser	2.50	2.49	2.67	2.57	2.50	2.60	2.46	2.49	2.59	2.51	2.54	0.07	2.61
Glu	8.66	8.55	9.31	8.98	8.68	9.05	8.44	8.63	9.05	8.65	8.80	0.28	3.16
Gly	2.06	2.05	2.15	2.10	2.03	2.09	2.01	2.06	2.13	2.04	2.07	0.05	2.23
Ala	2.06	2.04	2.19	2.15	2.06	2.11	2.02	2.08	2.18	2.05	2.09	0.06	2.90
Cys	0.45	0.41	0.45	0.45	0.47	0.45	0.40	0.40	0.41	0.44	0.43	0.03	6.27
Tyr	1.50	1.65	1.73	1.68	1.68	1.68	1.62	1.64	1.74	1.64	1.65	0.07	4.13
Pro	2.51	2.49	2.69	2.62	2.51	2.63	2.48	2.53	2.64	2.52	2.56	0.08	2.94

^1^ Determined by analysis and each value based on duplicate determinations. Abbreviations: SBM, soybean meal; GE, gross energy; CP, crude protein; EE, ether extract; CF, crude fiber; NDF, neutral detergent fiber; ADF, acid detergent fiber; CA, crude ash; NFE, nitrogen-free extract; PA, phytic acid; TI, trypsin inhibitor; EAAs, essential amino acids; Val, valine; Met, methionine; Ile, isoleucine; Leu, leucine; Thr, threonine; Phe, phenylalanine; Lys, lysine; His, histidine; Arg, arginine; Trp, tryptophan; NEAAs, non-essential amino acids; Asp, aspartic acid; Ser, serine; Glu, glutamic acid; Gly, glycine; Ala, alanine; Cys, cysteine; Tyr, tyrosine; Pro, proline; SD, standard deviation; CV, coefficient of variation.

**Table 3:** Composition and nutrient levels of 1 NFD and 10 tested SBM diets for medium-growing yellow-feathered chickens (as-fed basis).

Item	NFD	Tested SBM Diets									
		A1	A2	A3	A4	A5	A6	A7	A8	A9	A10
Ingredients, %											
SBM	-	38.26	45.26	38.26	38.26	38.26	38.26	45.26	38.26	38.26	45.26
Corn starch	63.81	19.02	16.70	19.02	19.02	19.02	19.02	16.70	19.02	19.02	16.70
Glucose ^1^	27.00	38.38	33.70	38.38	38.38	38.38	38.38	33.70	38.38	38.38	33.70
CaHPO_4_^1^	1.91	1.80	1.80	1.80	1.80	1.80	1.80	1.80	1.80	1.80	1.80
Limestone ^1^	1.16	1.30	1.30	1.30	1.30	1.30	1.30	1.30	1.30	1.30	1.30
NaCl ^1^	0.18	0.30	0.30	0.30	0.30	0.30	0.30	0.30	0.30	0.30	0.30
Cellulose ^1^	5.00	-	-	-	-	-	-	-	-	-	-
Choline chloride ^1^ (50%)	0.15	0.15	0.15	0.15	0.15	0.15	0.15	0.15	0.15	0.15	0.15
Sodium bicarbonate ^1^	0.15	0.15	0.15	0.15	0.15	0.15	0.15	0.15	0.15	0.15	0.15
Micronutrient premix ^1,2^	0.23	0.23	0.23	0.23	0.23	0.23	0.23	0.23	0.23	0.23	0.23
Antioxidant	0.01	0.01	0.01	0.01	0.01	0.01	0.01	0.01	0.01	0.01	0.01
TiO_2_	0.40	0.40	0.40	0.40	0.40	0.40	0.40	0.40	0.40	0.40	0.40
Total	100.00	100.00	100.00	100.00	100.00	100.00	100.00	100.00	100.00	100.00	100.00
Nutrient levels											
ME ^3^, kcal/kg	2848	2698	2647	2698	2698	2698	2698	2647	2698	2698	2647
CP ^4^, %	0.19	17.24	19.45	18.73	17.09	16.44	18.14	19.99	17.16	18.36	19.83
Lys ^4^, %	0.00	1.03	1.21	1.03	1.03	1.03	1.03	1.21	1.03	1.03	1.21
Met ^4^, %	0.00	0.23	0.27	0.23	0.23	0.23	0.23	0.27	0.23	0.23	0.27
Thr ^4^, %	0.00	0.65	0.77	0.65	0.65	0.65	0.65	0.77	0.65	0.65	0.77
Trp ^4^, %	0.00	0.22	0.26	0.22	0.22	0.22	0.22	0.26	0.22	0.22	0.26
Met + Cys ^4^, %	0.00	0.47	0.56	0.47	0.47	0.47	0.47	0.56	0.47	0.47	0.56
Ca ^3^, %	0.89	1.04	1.06	1.04	1.04	1.04	1.04	1.06	1.04	1.04	1.06
TP ^3^, %	0.38	0.65	0.70	0.65	0.65	0.65	0.65	0.70	0.65	0.65	0.70
Nonphytate P ^3^, %	0.37	0.41	0.43	0.41	0.41	0.41	0.41	0.43	0.41	0.41	0.43

^1^ Feed grade. ^2^ Provided per kilogram of diet: vitamin A 10,000 IU; cholecalciferol 3750 IU; vitamin E 27.5 IU; vitamin K 2.5 mg; vitamin B_1_ 2.5 mg; vitamin B_2_ 8.0 mg; vitamin B_6_ 3.75 mg; vitamin B_12_ 0.025 mg; calcium pantothenate 12.5 mg; niacin 45 mg; folate 125 mg; biotin 0.125 mg; zeolite 1347.8 mg; Cu (CuSO_4_·5H_2_O), 7 mg; Fe (FeSO_4_·H_2_O) 80 mg; Zn (ZnSO_4_·H_2_O) 55 mg; Mn (MnSO_4_·H_2_O) 60 mg; Se (Na_2_SeO_3_) 0.15 mg; I (Ca(IO_3_)_2_·H_2_O) 0.5 mg. ^3^ Calculated values. ^4^ Measured values based on duplicate determinations. Abbreviations: NFD, nitrogen-free diet; SBM, soybean meal; TiO_2_, titanium dioxide; ME, metabolizable energy; CP, crude protein; Lys, lysine; Met, methionine; Thr, threonine; Trp, tryptophan; Cys, cysteine; Ca, calcium; TP, total phosphorus.

**Table 4:** AIAADs of SBMs from different sources for medium-growing yellow-feathered chickens.

Item (%)	SBM Sources										Mean	Pooled SE	CV (%)	*p*-Value
	A1	A2	A3	A4	A5	A6	A7	A8	A9	A10				
EAAs														
Val	83.6 ^bcd^	82.0 ^de^	84.9 ^abc^	82.9 ^cd^	80.0 ^e^	81.0 ^de^	85.1 ^abc^	84.9 ^abc^	86.6 ^a^	85.6 ^ab^	83.7	0.9	2.43	<0.001
Met	89.3 ^a^	87.7 ^ab^	87.3 ^abc^	84.9 ^cd^	86.0 ^bc^	87.2 ^abc^	86.2 ^bc^	85.0 ^cd^	86.3 ^bc^	82.9 ^d^	86.3	0.9	1.94	0.003
Ile	85.5 ^bc^	85.1 ^bc^	87.7 ^a^	84.3 ^c^	85.3 ^bc^	85.8 ^b^	85.6 ^bc^	86.4 ^ab^	87.7 ^a^	85.7 ^b^	85.9	0.5	1.20	<0.001
Leu	87.0 ^d^	87.0 ^d^	89.9 ^ab^	88.0 ^cd^	89.6 ^ab^	90.4 ^a^	89.8 ^ab^	88.9 ^bc^	89.7 ^ab^	89.5 ^ab^	89.0	0.5	1.31	<0.001
Thr	78.6 ^ab^	77.4 ^bc^	80.6 ^a^	74.7 ^cd^	76.5 ^bcd^	74.5 ^d^	77.2 ^bcd^	74.7 ^cd^	75.5 ^cd^	80.3 ^a^	77.0	1.0	2.78	<0.001
Phe	83.4 ^d^	84.5 ^d^	86.4 ^bc^	84.7 ^d^	85.2 ^cd^	88.5 ^a^	88.7 ^a^	88.9 ^a^	89.7 ^a^	88.1 ^ab^	86.8	0.6	2.44	<0.001
Lys	85.5 ^bcd^	82.9 ^e^	86.2 ^ab^	84.6 ^cd^	84.3 ^d^	85.8 ^bc^	87.4 ^a^	85.5 ^bcd^	86.6 ^ab^	85.4 ^bcd^	85.4	0.5	1.40	<0.001
His	84.4 ^bc^	80.9 ^de^	84.7 ^ab^	80.1 ^e^	81.8 ^cde^	83.6 ^bcd^	87.1 ^a^	86.1 ^ab^	85.0 ^ab^	84.3 ^bc^	83.8	1.0	2.54	<0.001
Arg	90.1 ^bc^	88.7 ^de^	90.5 ^abc^	88.7 ^de^	89.0 ^de^	90.0 ^bcd^	90.9 ^ab^	89.8 ^cd^	91.4 ^a^	91.2 ^a^	90.0	0.4	1.05	<0.001
Trp	76.3 ^e^	80.9 ^b^	85.4 ^a^	81.0 ^b^	77.7 ^cde^	76.7 ^de^	79.5 ^bcd^	80.4 ^bc^	77.0 ^de^	79.3 ^bcde^	79.4	1.1	3.27	<0.001
NEAAs														
Asp	80.0 ^d^	80.2 ^d^	84.1 ^bc^	82.3 ^cd^	83.4 ^cd^	85.4 ^abc^	84.6 ^bc^	87.7 ^ab^	85.5 ^abc^	88.2 ^a^	84.1	1.2	3.13	<0.001
Ser	83.6 ^de^	81.9 ^f^	85.7 ^abc^	82.3 ^ef^	85.1 ^bcd^	83.6 ^de^	84.4 ^cd^	81.9 ^f^	86.5 ^ab^	86.9 ^a^	84.2	0.5	2.08	<0.001
Glu	87.9 ^g^	90.4 ^cdef^	92.7 ^a^	91.4 ^abcd^	90.9 ^bcde^	92.1 ^ab^	91.4 ^abc^	89.8 ^ef^	89.2 ^fg^	89.9 ^def^	90.6	0.5	1.50	<0.001
Gly	78.4 ^bc^	76.9 ^bc^	77.8 ^bc^	76.2 ^c^	75.7 ^c^	78.4 ^bc^	78.1 ^bc^	78.4 ^bc^	79.6 ^b^	83.0 ^a^	78.3	1.0	2.46	0.001
Ala	80.4	79.1	82.0	78.8	78.4	80.9	82.7	79.5	79.6	82.7	80.4	1.2	1.89	0.095
Cys	72.5	71.5	72.3	68.9	71.0	72.6	74.0	71.8	72.9	73.0	72.1	1.6	1.83	0.631
Tyr	84.6	82.6	85.7	83.2	81.6	82.2	83.6	82.5	83.1	83.7	83.3	1.0	1.37	0.219
Pro	73.8 ^de^	75.2 ^cd^	76.3 ^abcd^	71.1 ^e^	73.4 ^de^	73.7 ^de^	76.0 ^bcd^	79.4 ^ab^	79.7 ^a^	78.6 ^abc^	75.7	1.2	3.56	<0.001

Values represent the means ± standard deviations of 5–6 replicate cages (4 birds per replicate cage) (*n* = 5–6); Different lowercase letters on the shoulder of peer data indicate significant differences (*p* < 0.05), while the same or no letters indicate no significant differences (*p* > 0.05). Abbreviations: SBMs, soybean meals; AIAADs, apparent ileal amino acid digestibilities; EAAs, essential amino acids; Val, valine; Met, methionine; Ile, isoleucine; Leu, leucine; Thr, threonine; Phe, phenylalanine; Lys, lysine; His, histidine; Arg, arginine; Trp, tryptophan; NEAAs, non-essential amino acids; Asp, aspartic acid; Ser, serine; Glu, glutamic acid; Gly, glycine; Ala, alanine; Cys, cysteine; Tyr, tyrosine; Pro, proline; CV, coefficient of variation.

**Table 5:** SIAADs of SBMs from different sources for medium-growing yellow-feathered chickens.

Item (%)	SBM sources										Mean	Pooled SE	CV (%)	*p*-Value
	A1	A2	A3	A4	A5	A6	A7	A8	A9	A10				
EAAs														
Val	89.7 ^bcd^	87.8 ^cde^	91.2 ^ab^	90.1 ^abc^	86.8 ^e^	87.4 ^de^	90.6 ^ab^	91.4 ^ab^	92.3 ^a^	91.0 ^ab^	89.8	0.9	1.98	<0.001
Met	92.9 ^a^	91.4 ^ab^	91.2 ^ab^	89.4 ^b^	90.2 ^ab^	91.1 ^ab^	89.5 ^b^	89.1 ^b^	89.7 ^b^	86.2 ^c^	90.1	0.9	1.89	0.002
Ile	89.6 ^cd^	88.9 ^d^	91.8 ^a^	89.1 ^d^	89.8 ^cd^	90.1 ^bcd^	89.2 ^d^	90.7 ^abc^	91.4 ^ab^	89.3 ^cd^	90.0	0.5	1.06	0.001
Leu	91.3 ^d^	91.0 ^d^	94.2 ^abc^	93.1 ^c^	94.4 ^ab^	94.8 ^a^	93.6 ^abc^	93.5 ^bc^	93.7 ^abc^	93.2 ^bc^	93.3	0.5	1.26	<0.001
Thr	86.6 ^abc^	85.0 ^bcd^	88.8 ^a^	84.2 ^cd^	85.5 ^bcd^	82.8 ^d^	84.3 ^cd^	83.5 ^cd^	83.1 ^d^	87.4 ^ab^	85.1	1.0	2.19	0.001
Phe	88.7 ^f^	89.4 ^ef^	91.8 ^cd^	91.0 ^de^	91.2 ^de^	94.0 ^ab^	93.3 ^abc^	94.6 ^a^	94.6 ^a^	92.6 ^bcd^	92.1	0.6	2.14	<0.001
Lys	88.9 ^bc^	86.3 ^d^	89.8 ^ab^	88.6 ^bc^	88.2 ^c^	89.4 ^abc^	90.4 ^a^	89.2 ^abc^	89.8 ^ab^	88.5 ^bc^	88.9	0.5	1.22	<0.001
His	87.1 ^abc^	83.5 ^d^	87.4 ^abc^	83.2 ^d^	84.8 ^cd^	86.4 ^bc^	89.5 ^a^	89.0 ^ab^	87.5 ^abc^	86.6 ^bc^	86.5	1.0	2.32	<0.001
Arg	93.8 ^bcd^	92.3 ^e^	94.3 ^abc^	93.1 ^de^	93.2 ^cde^	93.8 ^abcd^	94.1 ^abc^	93.8 ^bcd^	94.8 ^a^	94.5 ^ab^	93.8	0.4	0.75	0.001
Trp	81.8 ^d^	85.2 ^bc^	89.8 ^a^	85.8 ^b^	83.8 ^bcd^	82.5 ^cd^	84.3 ^bcd^	86.1 ^b^	82.2 ^cd^	83.5 ^bcd^	84.5	1.1	2.67	<0.001
NEAAs														
Asp	84.3 ^d^	84.3 ^d^	88.5 ^bc^	87.4 ^cd^	88.3 ^bc^	89.9 ^abc^	88.5 ^bc^	92.3 ^a^	89.5 ^abc^	92.0 ^ab^	88.5	1.2	2.91	<0.001
Ser	89.8 ^b^	87.8 ^c^	92.1 ^a^	89.6 ^b^	92.1 ^a^	90.0 ^b^	89.8 ^b^	88.7 ^bc^	92.3 ^a^	92.3 ^a^	90.5	0.5	1.72	<0.001
Glu	91.2 ^e^	93.5 ^cd^	96.0 ^a^	95.3 ^ab^	94.6 ^abc^	95.5 ^ab^	94.3 ^bc^	93.4 ^cd^	92.2 ^de^	92.8 ^d^	93.9	0.5	1.56	<0.001
Gly	83.3 ^bc^	81.6 ^bc^	82.9 ^bc^	82.0 ^bc^	81.3 ^c^	83.5 ^bc^	82.4 ^bc^	83.7 ^bc^	84.1 ^b^	87.2 ^a^	83.2	1.0	1.92	0.007
Ala	85.6	83.9	87.3	84.9	84.2	86.3	87.3	85.1	84.4	87.2	85.6	1.2	1.47	0.311
Cys	81.6	80.7	82.4	80.2	81.5	82.6	82.7	82.2	82.0	81.5	81.7	1.6	0.94	0.980
Tyr	87.3	85.1	88.4	86.4	84.6	85.0	85.9	85.4	85.5	85.9	86.0	1.0	1.27	0.276
Pro	82.3 ^de^	83.2 ^cde^	85.0 ^bcd^	81.1 ^e^	83.2 ^cde^	82.7 ^cde^	83.6 ^cde^	88.8 ^a^	88.2 ^ab^	86.1 ^abc^	84.4	1.2	2.87	<0.001

Values represent the means ± standard deviations of 5–6 replicate cages (4 birds per replicate cage) (*n* = 5–6); Different lowercase letters on the shoulder of peer data indicate significant differences (*p* < 0.05), while the same or no letters indicate no significant differences (*p* > 0.05). Abbreviations: SIAADs, standardized ileal amino acid digestibilities; SBMs, soybean meals; EAAs, essential amino acids; Val, valine; Met, methionine; Ile, isoleucine; Leu, leucine; Thr, threonine; Phe, phenylalanine; Lys, lysine; His, histidine; Arg, arginine; Trp, tryptophan; NEAAs, non-essential amino acids; Asp, aspartic acid; Ser, serine; Glu, glutamic acid; Gly, glycine; Ala, alanine; Cys, cysteine; Tyr, tyrosine; Pro, proline; CV, coefficient of variation.

**Table 6:** Correlations between chemical compositions (air-dry basis) and SIAADs of SBMs for medium-growing yellow-feathered chickens.

Item (%)	GE (MJ/kg)	CP (%)	EE (%)	CF (%)	NDF (%)	ADF (%)	CA (%)	PA (g/kg)	NFE (%)	TI (g/kg)
EAAs										
Val	0.589	0.201	−0.650	−0.243	−0.462	−0.370	0.168	0.074	0.586	−0.111
Met	−0.549	0.231	0.638	−0.364	0.008	−0.210	−0.565	0.306	−0.145	−0.354
Ile	0.221	0.889 **	−0.366	−0.876 **	−0.524	−0.813 **	0.428	−0.087	0.319	−0.335
Leu	0.097	0.612	−0.304	−0.299	−0.415	−0.471	0.894 **	−0.686 *	−0.364	−0.100
Thr	−0.145	−0.256	0.488	−0.123	0.497	0.184	−0.311	−0.305	0.216	0.282
Phe	0.504	0.513	−0.846 **	−0.125	−0.668 *	−0.451	0.821 **	−0.299	−0.030	−0.319
Lys	0.299	0.494	−0.341	−0.294	−0.744 *	−0.632	0.499	−0.420	−0.042	−0.188
His	0.399	0.254	−0.455	−0.115	−0.738 *	−0.479	0.376	−0.263	0.181	−0.181
Arg	0.677 *	0.405	−0.545	−0.317	−0.619	−0.571	0.459	−0.241	0.334	−0.110
Trp	−0.361	0.154	0.050	−0.259	0.241	0.013	0.072	−0.457	−0.037	0.078
NEAAs										
Asp	0.622	0.342	−0.724 *	−0.098	−0.486	−0.339	0.858 **	−0.453	0.163	−0.085
Ser	0.265	0.324	−0.129	−0.351	−0.065	−0.318	0.494	−0.329	0.080	0.063
Glu	−0.485	0.297	0.139	−0.081	0.138	−0.006	0.400	−0.797 *	−0.672 *	−0.186
Gly	0.905 **	−0.037	−0.536	0.031	−0.275	−0.142	0.205	−0.078	0.566	−0.011
Ala	0.208	−0.087	−0.022	0.151	−0.149	−0.037	0.129	−0.753 *	−0.127	−0.302
Cys	0.161	0.526	−0.298	−0.230	−0.710 *	−0.571	0.544	−0.584	−0.251	−0.229
Tyr	0.005	0.128	0.273	−0.412	−0.051	−0.279	−0.347	−0.191	0.328	−0.250
Pro	0.628	0.383	−0.864 **	−0.325	−0.461	−0.400	0.515	0.168	0.591	0.053

* Represents a significant difference (*p* < 0.05); ** represents a highly significant difference (*p* < 0.01). The SIAAD values of 9 tested soybean meals (*n* = 9) were used for correlation (r) analysis. Abbreviations: SIAADs, standardized ileal amino acid digestibilities; SBMs, soybean meals; GE, gross energy; CP, crude protein; EE, ether extract; CF, crude fiber; NDF, neutral detergent fiber; ADF, acid detergent fiber; CA, crude ash; PA, phytic acid; NFE, nitrogen-free extract; TI, trypsin inhibitor; EAAs, essential amino acids; Val, valine; Met, methionine; Ile, isoleucine; Leu, leucine; Thr, threonine; Phe, phenylalanine; Lys, lysine; His, histidine; Arg, arginine; Trp, tryptophan; NEAAs, non-essential amino acids; Asp, aspartic acid; Ser, serine; Glu, glutamic acid; Gly, glycine; Ala, alanine; Cys, cysteine; Tyr, tyrosine; Pro, proline.

**Table 7:** Correlations between EAAs (air-dry basis) and SIAADs of SBMs for medium-growing yellow-feathered chickens.

Items (%)	Val (%)	Met (%)	Ile (%)	Leu (%)	Thr (%)	Phe (%)	Lys (%)	His (%)	Arg (%)	Trp (%)
EAAs										
Val	0.239	−0.074	0.153	0.241	0.266	0.025	0.333	0.319	0.358	−0.145
Met	0.327	0.010	0.259	0.181	0.163	0.321	0.434	0.303	−0.022	0.129
Ile	0.889 **	0.572	0.859 **	0.900 **	0.902 **	0.651	0.718 *	0.905 **	0.895 **	0.436
Leu	0.410	0.705 *	0.470	0.503	0.581	0.192	0.171	0.473	0.654	0.553
Thr	0.106	0.238	0.088	0.087	0.057	0.117	0.210	0.046	−0.003	−0.420
Phe	0.229	0.234	0.266	0.335	0.385	0.060	0.027	0.309	0.527	0.479
Lys	0.361	0.372	0.272	0.320	0.470	−0.070	0.479	0.538	0.500	0.309
His	0.058	−0.027	−0.043	0.025	0.142	−0.309	0.224	0.246	0.176	0.112
Arg	0.412	0.452	0.331	0.388	0.534	−0.004	0.541	0.548	0.589	0.235
Trp	0.280	0.059	0.348	0.395	0.223	0.490	0.013	0.167	0.274	−0.209
NEAAs										
Asp	0.102	0.343	0.154	0.224	0.286	−0.086	−0.077	0.174	0.386	0.416
Ser	0.437	0.730 *	0.427	0.457	0.563	0.167	0.411	0.481	0.626	0.094
Glu	0.287	0.430	0.415	0.417	0.328	0.451	−0.054	0.198	0.386	0.248
Gly	0.037	0.220	0.016	0.047	0.139	−0.162	0.161	0.084	0.154	0.176
Ala	0.043	0.281	0.026	0.048	0.117	−0.121	0.183	0.102	0.133	0.007
Cys	0.344	0.378	0.307	0.341	0.449	0.022	0.352	0.481	0.469	0.460
Tyr	0.436	0.228	0.338	0.344	0.353	0.263	0.637	0.450	0.255	−0.166
Pro	0.231	−0.023	0.227	0.319	0.299	0.123	0.070	0.265	0.417	0.113

* Represents a significant difference (*p* < 0.05); ** represents a highly significant difference (*p* < 0.01). The SIAAD values of 9 tested soybean meals (*n* = 9) were used for correlation (r) analysis. Abbreviations: EAAs, essential amino acids; SIAADs, standardized ileal amino acid digestibilities; SBMs, soybean meals; Val, valine; Met, methionine; Ile, isoleucine; Leu, leucine; Thr, threonine; Phe, phenylalanine; Lys, lysine; His, histidine; Arg, arginine; Trp, tryptophan; NEAAs, non-essential amino acids; Asp, aspartic acid; Ser, serine; Glu, glutamic acid; Gly, glycine; Ala, alanine; Cys, cysteine; Tyr, tyrosine; Pro, proline.

**Table 8:** Correlations between NEAAs (air-dry basis) and SIAADs of SBMs for medium-growing yellow-feathered chickens.

Items (%)	Asp (%)	Ser (%)	Glu (%)	Gly (%)	Ala (%)	Cys (%)	Tyr (%)	Pro (%)
EAAs								
Val	0.284	0.188	0.191	0.374	0.405	−0.509	0.123	0.287
Met	0.222	0.210	0.222	0.272	0.165	0.234	−0.253	0.157
Ile	0.912 **	0.794 *	0.835 **	0.875 **	0.939 **	0.052	0.611	0.864 **
Leu	0.483	0.477	0.506	0.269	0.437	0.268	0.641	0.539
Thr	0.122	0.223	0.173	0.128	0.075	0.487	−0.132	0.077
Phe	0.309	0.233	0.262	0.222	0.354	−0.387	0.550	0.388
Lys	0.393	0.344	0.369	0.304	0.413	−0.097	0.150	0.447
His	0.096	0.005	0.023	0.069	0.142	−0.427	−0.103	0.131
Arg	0.456	0.416	0.438	0.420	0.513	−0.041	0.226	0.505
Trp	0.350	0.375	0.323	0.340	0.321	−0.067	0.390	0.322
NEAAs								
Asp	0.203	0.147	0.170	0.076	0.206	−0.134	0.423	0.254
Ser	0.478	0.488	0.515	0.363	0.502	0.510	0.464	0.479
Glu	0.356	0.469	0.426	0.205	0.249	0.259	0.571	0.419
Gly	0.086	0.106	0.098	0.105	0.101	−0.034	−0.032	0.142
Ala	0.106	0.249	0.185	0.047	0.040	0.100	−0.074	0.215
Cys	0.387	0.374	0.383	0.269	0.356	−0.097	0.219	0.467
Tyr	0.428	0.465	0.429	0.484	0.405	0.185	−0.156	0.399
Pro	0.294	0.128	0.167	0.327	0.410	−0.512	0.418	0.265

* Represents a significant difference (*p* < 0.05); ** represents a highly significant difference (*p* < 0.01). The SIAAD values of 9 tested soybean meals (*n* = 9) were used for correlation (r) analysis. Abbreviations: NEAAs, non-essential amino acids; SIAADs, standardized ileal amino acid digestibilities; SBMs, soybean meals; EAAs, essential amino acids; Val, valine; Met, methionine; Ile, isoleucine; Leu, leucine; Thr, threonine; Phe, phenylalanine; Lys, lysine; His, histidine; Arg, arginine; Trp, tryptophan; Asp, aspartic acid; Ser, serine; Glu, glutamic acid; Gly, glycine; Ala, alanine; Cys, cysteine; Tyr, tyrosine; Pro, proline.

**Table 9:** Established predicting models of SIAADs in SBMs for medium-growing yellow-feathered chickens. based on their chemical compositions and AA profiles (air-dry basis) *.

Predicting Models	*R* ^2^	RMSE	*p*-Value
EAAs (%)			
SID Met = 63.57 + 0.98 CP + 6.96 EE − 1.41 CA − 38.24 Cys	0.953	0.010	0.006
SID Ile = 65.44 − 0.12 ADF + 11.26 Ala	0.920	0.331	0.001
SID Leu = 68.8 + 1.79 CA − 0.18 NFE + 6.54 Ser	0.972	0.001	0.0003
SID Phe = 84.30 − 4.57 EE + 1.92 CA	0.909	0.752	0.001
SID Lys = 108.98 − 0.34 NDF − 0.28 PA − 21.67 Trp	0.886	0.248	0.009
SID His = 130.69 − 0.63 NDF − 0.35 PA − 55.77 Trp	0.980	0.340	0.0001
SID Arg = 52.79 + 1.78 GE − 5.63 Phe + 6.94 Arg	0.840	0.347	0.020
NEAAs (%)			
SID Asp = −106.28 + 11.17 GE + 3.20 CA − 0.42 PA − 29.60 Met	0.965	0.749	0.004
SID Ser = 74.28 − 2.82 EE − 7.94 Ser + 124.82 Met − 57.18 Trp	0.956	0.349	0.006
SID Glu = 81.27 − 0.55 PA + 7.19 Phe	0.831	0.540	0.005
SID Gly = −115.93 + 11.50 GE	0.820	0.017	0.001
SID Ala = 90.70 − 0.46 PA	0.567	0.972	0.019
SID Cys = 104.05 − 1.22 GE − 0.14 NDF − 0.15 PA − 0.08 TI + 1.21 Lys	0.993	0.0004	0.002
SID Tyr = 67.11 − 0.20 PA + 12.36 Lys − 31.57 Trp	0.821	0.649	0.026
SID Pro = 94.23 − 7.71 EE	0.747	0.004	0.003

* The SIAAD values of 9 tested soybean meals (*n* = 9) were used to construct the SIAADs-predicting models. Abbreviations: SIAADs, standardized ileal amino acid digestibilities; SBMs, soybean meals; AA, amino acid; SID, standardized ileal digestibility; CP, crude protein (%); EE, ether extract (%); CA, crude ash (%); ADF, acid detergent fiber (%); NFE, nitrogen-free extract (%); NDF, neutral detergent fiber (%); PA, phytic acid (g/kg); GE, gross energy (MJ/kg); TI, trypsin inhibitor (g/kg); EAAs, essential amino acids; Met, methionine; Cys, cysteine; Ile, isoleucine; Ala, alanine; Leu, leucine; Ser, serine; Phe, phenylalanine; Lys, lysine; Trp, tryptophan; His, histidine; Arg, arginine; NEAAs, non-essential amino acids; Asp, aspartic acid; Glu, glutamic acid; Gly, glycine; Tyr, tyrosine; Pro, proline; *R*^2^, coefficient of determination; RMSE, root mean square error.

**Table 10:** Verification of the accuracies of predicting models for SIAADs in SBMs for medium-growing yellow-feathered chickens.

Item (%)	Determined Value ± SD of SIAAD in SBM A4 ^1^ (%)	Predicted Value of SIAAD in SBM A4 (%)	Difference Value ^2^ (%)	Accuracy Evaluation
SID Met	89.4 ± 1.7	91.1	1.7	Accurate
SID Ile	89.1 ± 1.4	89.0	0.1	Accurate
SID Leu	93.1 ± 0.6	93.7	0.6	Accurate
SID Phe	91.0 ± 1.4	92.2	1.2	Accurate
SID Lys	88.6 ± 1.1	89.7	1.1	Accurate
SID His	83.2 ± 2.1	87.7	4.5	Inaccurate
SID Arg	93.1 ± 0.8	93.9	0.8	Accurate
SID Asp	87.4 ± 1.8	89.1	1.7	Accurate
SID Ser	89.6 ± 2.1	91.1	1.5	Accurate
SID Glu	95.3 ± 0.9	94.6	0.7	Accurate
SID Gly	82.0 ± 1.2	83.1	1.1	Accurate
SID Ala	84.9 ± 1.4	86.2	1.3	Accurate
SID Cys	80.2 ± 2.7	82.4	2.2	Accurate
SID Tyr	86.4 ± 0.3	86.6	0.2	Accurate
SID Pro	81.1 ± 2.1	86.8	5.7	Inaccurate

^1^ The determined value is the mean ± standard deviation (SD) of 5–6 replicate values (*n* = 5–6). ^2^ The difference value equals the deviation between determined and predicted values. Abbreviations: SIAADs, standardized ileal amino acid digestibilities; SBMs, soybean meals; SID, standardized ileal digestibility; Met, methionine; Ile, isoleucine; Leu, leucine; Phe, phenylalanine; Lys, lysine; His, histidine; Arg, arginine; Asp, aspartic acid; Ser, serine; Glu, glutamic acid; Gly, glycine; Ala, alanine; Cys, cysteine; Tyr, tyrosine; Pro, proline.

## Data Availability

The data presented in this study are available in the article.
